# A Driving System for Fast and Precise Gray-Scale Response Based on Amplitude–Frequency Mixed Modulation in TFT Electrowetting Displays

**DOI:** 10.3390/mi10110732

**Published:** 2019-10-29

**Authors:** Zichuan Yi, Linwei Liu, Li Wang, Wei Li, Lingling Shui, Guofu Zhou

**Affiliations:** 1 University of Electronic Science and Technology of China, Zhongshan Institute, Zhongshan 528402, China; yizichuan@163.com (Z.Y.); Shuill@m.scnu.edu.cn (L.S.); 2 Shenzhen Guohua Optoelectronics Tech. Co., Ltd., Shenzhen 518110, China; creekxi@163.com (L.W.); wei.li@guohua-oet.com (W.L.); guofu.zhou@m.scnu.edu.cn (G.Z.); 3 Institute of Electronic Paper Displays, South China Academy of Advanced Optoelectronics, South China Normal University, Guangzhou 510006, China

**Keywords:** electrowetting display, gray scale, response time, gray-scale oscillation, amplitude–frequency mixed modulation

## Abstract

The gray-scale display which is driven by PWM (pulse width modulation) in TFT (thin film transistor) electrowetting displays (EWDs) has some shortcomings, such as large amplitude of oil oscillation in pixels and slow response speed for displaying gray scale. In this paper, an amplitude–frequency mixed modulation driving system is proposed to improve the response speed of driving gray scale and enhance the oil stability when the gray scale is displayed. In the initial stage of the driving process, the oil is driven by a high voltage to close to the target luminance, and the driving voltage is then decreased to stabilize the oil. The electrowetting hysteresis curve was used to calculate the relation model between the driving voltage and the luminance of the pixel in the system, and the driving voltage value of the pixel at each driving stage was then set to make the oil precisely and rapidly stabilize at the target luminance value. In the output driving platform, the amplitude–frequency mixed modulation is realized based on the source IC, which was used to realize digital-to-analog conversion. An 8 inch EWD was tested using an Admesy colorimeter, and the experimental results show that the pixel response time is reduced by 70% and the gray-scale oscillation is reduced by 80%.

## 1. Introduction

Electrowetting displays (EWDs) have a paper-like display characteristic and excellent visual performance under strong light environment [1,2]. They have attracted the attention of many researchers and business people. Compared with traditional liquid crystal displays (LCD), the EWD has many advantages including a higher contrast ratio in strong light, lower power consumption, and the fact that it can realize a kind of paper-like display [3,4]. The principle of EWDs is an optical switch which is realized by controlling the colored oil distribution in the pixel with applying a certain voltage. When the colored oil is driven from a corner of the pixel, the color of the pixel substrate is displayed. When the voltage is evacuated, the oil is spread over the whole pixel to show the color of the oil. Then, the gray-scale display can be realized by controlling the area of oil in the pixel [5,6]. Hays designed and implemented the first EWD device in 2003 [7], and the main structure of this EWD has continued to be used until today [8,9].

At present, EWDs are still plagued by several problems which seriously affect the display performance, including hysteresis [10,11], oil splitting [12,13], and gray-scale display stability [13,14]. Recently, a driving waveform of an AC common driver was proposed, which can reduce the distance between the rising and falling curves of oil hysteresis [8]. This can improve the oil splitting performance and improve the hysteresis phenomenon of the oil. However, the inhomogeneity of gray-scale display still exists, and the pulse width modulation (PWM) used by the system cannot make fine adjustments for the gray-scale display. At the same time, a new technology which is used to improve the oil splitting problem according to the distribution characteristics of oil in the pixel is proposed [13]. However, the oil splitting is not only related to the distribution of the oil, but also the driving waveform. In addition, we used the industrialized electrophoretic electronic paper driving chip to drive EWDs, and a four-level gray-scale display is realized by PWM in the EWD [15], but the oil has a sharp oscillation in the pixel, which affects the life of EWDs. Then, the effect of voltage curve slope on the luminance of EWDs has been studied by our research team [16], and EWDs can achieve different luminance values with different voltage slope values. However, the driving waveform of the driving algorithm with a slope is difficult to implement in the thin film transistor (TFT)-EWD. A 16-level gray-scale driving waveform has been proposed to drive a TFT-EWD [17]. However, the PWM is used in the system, and the conversion between two frames requires 28 ms, which can cause serious flicker for human vision. Worse still, the oil oscillation frequency is too high, which has a bad effect on the display performance of the EWD. In the field of driving LCD, overdrive technology has been widely used to accelerate the response speed of LCDs [18], and this overdrive technology can be also used to improve the response speed of EWDs.

Hence, a TFT-EWD driving system based on amplitude–frequency mixed modulation is proposed in this paper. The high-speed hardware of digital to analog converter (DAC) was used to modulate the amplitude and display timing to accurately control the oil in the pixel. The system can realize a fast response speed for gray-scale display in EWDs, and a stable oil distribution can be obtained quickly. A set of driving waveforms was designed to test the system which can solve the problems caused by the traditional PWM method for driving EWDs.

## 2. Electrowetting Display (EWD) Driving Principle

### 2.1. Structure of EWD

**Figure 1:**
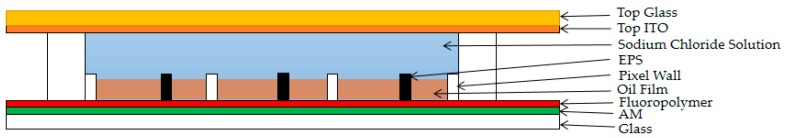
Electrowetting display (EWD) structure.

**Figure 2:**
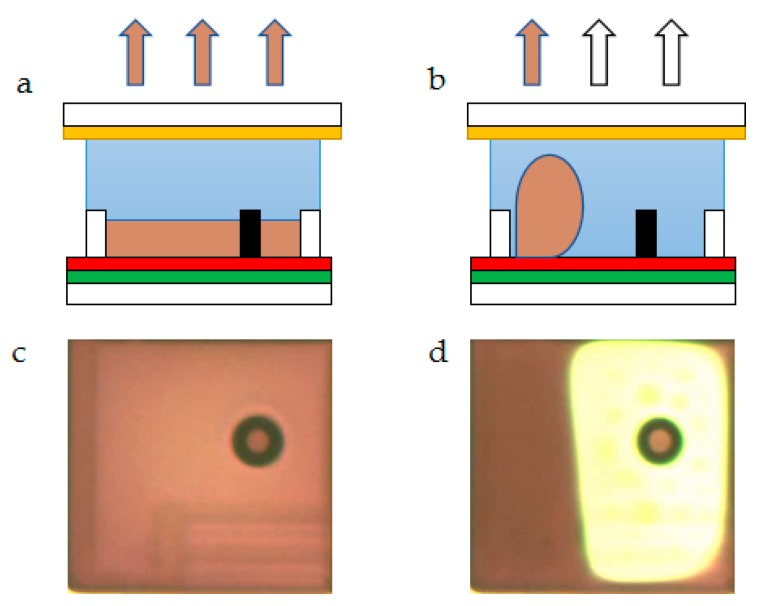
EWD principle. (**a**) The oil is spread over the entire pixel when the pixel is turned off. (**b**) The oil is pushed to a corner in the pixel when the pixel is turned on. (**c**) The top view when the pixel is turned off. (**d**) The top view when the pixel is turned on.

As shown in Figure 1, an EWD device includes top ITO (indium tin oxide), sodium chloride solution, sealant, active matrix (AM) substrate, a hydrophobic layer, oil, pixel wall, and EPS (extra pinning structure) [19]. The EPS is used to automatically fill the entire pixel space by capillary action when the oil requires filling. The EPS is made of hydrophobic insulating material, and its height is the same as the pixel wall. It is designed at the center of the pixel, as shown in Figure 2a. The pixel is turned off when no voltage is applied, and the color of the oil is displayed on the pixel. The top view is shown in Figure 2c, and the oil is pushed to a corner in the pixel due to the electrostatic force when the voltage is applied, and the pixel is then turned on, as shown in Figure 2b. At this time, the reflected light is the color of the pixel substrate and the color of a small portion of the oil, and the top view is shown in Figure 2d. During the pixel opening and closing process, the oil in the pixel will exhibit different aperture ratios at the same voltage value, which is an important feature of EWDs called hysteresis.

### 2.2. Hysteresis of EWDs

The hysteresis effect of electrowetting has been discovered as early as 2006. In electrowetting, the droplet requires a force which is greater than the threshold force for moving. The contact angle of hysteresis caused by random pinning forces is a major obstacle for moving liquid on solid surfaces [20]. The hysteresis effect and response time are key factors for the display effect of EWDs. The hysteresis phenomenon is as follows: when the driving voltage is increased, the oil is gradually pushed to one side in the pixel, and the luminance of the pixel is also increased. However, in the process of voltage reduction, there is a difference in pixel luminance at the same driving voltage value.

Both the voltage rising curve and the falling curve satisfy the electrowetting Young–Lippmann equation, as shown in Equation (1).

(1) $$\cos\theta =\cos\theta_{0}+\frac{\varepsilon_{0}\varepsilon_{r}V^{2}}{2d\gamma_{LV}}$$

In Equation (1), θ is the solid–liquid interface contact angle, $\theta_{0}$ is the solid–liquid interface equilibrium contact angle, $\varepsilon_{0}$ is the vacuum dielectric constant, and $\varepsilon_{r}$, *d* are the dielectric constant and thickness of the hydrophobic layer, respectively. *V* is the applied voltage on the pixel. In the amplitude–frequency mixed modulation scheme, the hysteresis characteristic curve of the EWD is used to design the driving voltage amplitude in the driving waveform.

### 2.3. Principle of Amplitude–Frequency Mixed Modulation

**Figure 3:**
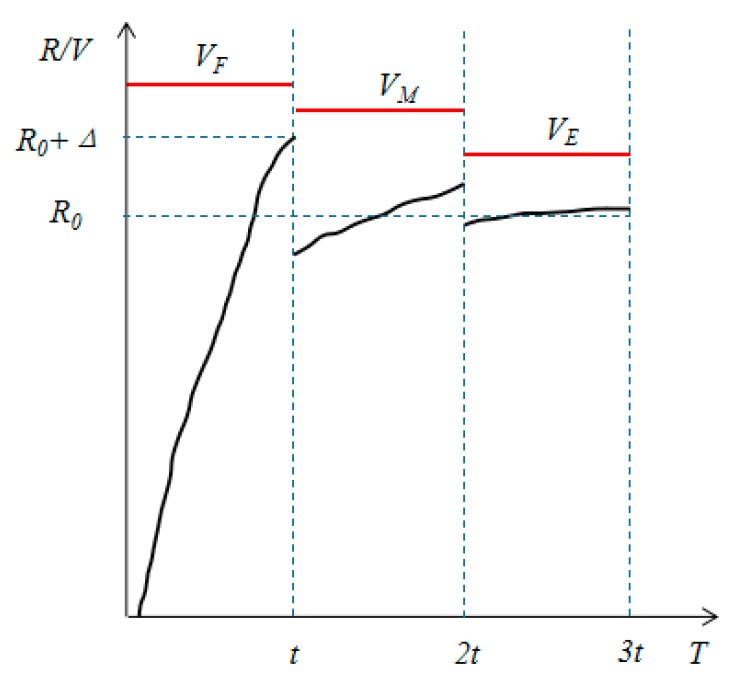
Design of the driving waveform for amplitude–frequency mixed modulation.

In the amplitude–frequency mixed modulation scheme, the driving waveform is converted into multiple subframes, and then the luminance is precisely controlled. As shown in Figure 3, $V_{F}$, $V_{M}$, and $V_{E}$ are the voltages of three subframes in a driving waveform respectively, which can form a complete gray-scale conversion process. $V_{F}$ is the initial voltage at which the pixel can be turned on, and has a relatively large voltage value, which can improve the response speed of EWDs. $V_{M}$ is the voltage for controlling the oscillation of the pixel luminance and $V_{E}$ is the driving voltage of the target gray scale. The oil is pushed to a corner of the pixel when the voltage $V_{F}$ is applied for a time *t*, and the surface energy of the oil is reduced and the kinetic energy is increased. The electric field force cannot maintain the oil’s state when the applied voltage is reduced to $V_{M}$, and the oil has an impact on the contact surface by increasing kinetic energy at this time [21]. At 2*t* the driving voltage is reduced to $V_{E}$, and the oil can reach a balance between interface energy and kinetic energy.

**Figure 4:**
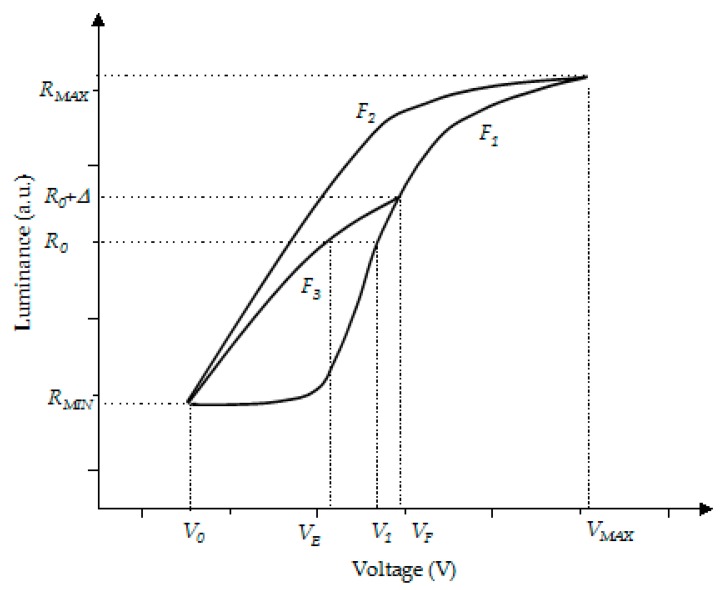
Design of driving waveform voltage amplitude in amplitude–frequency mixed modulation.

The hysteresis curve, which is used in the voltage amplitude design of the amplitude–frequency mixed modulation, is shown in Figure 4. When the voltage is slowly increased from $V_{0}$ to $V_{MAX}$, the luminance response curve is $F_{1}\left(V\right)$. The luminance response curve of the voltage falling from $V_{MAX}$ to $V_{0}$ is called $F_{2}\left(V\right)$ However, when the maximum driving voltage of the pixel is reduced to $V_{F}$, the rising edge of the response curve becomes a part of $F_{1}\left(V\right)$, but the falling edge of the response curve is $F_{3}\left(V\right)$. In Figure 4, $V_{0}$ is the minimum driving voltage of the oil, which is 0 V, and $V_{MAX}$ is the maximum voltage for driving oil. $V_{1}$ is the voltage at which the oil can reach the target *R_0_* on $F_{1}\left(V\right)$, and $V_{E}$ is the voltage at which the oil can reach the target *R_0_* on $F_{3}\left(V\right)$. *R_0_* is the target luminance in the design, Δ is the permissible jitter range of the fixed gray-scale luminance, *R_MIN_* is the minimum luminance of the pixel, and *R_MAX_* is the maximum luminance of the pixel.

Principle of the amplitude–frequency mixed modulation: The maximum voltage which is set in the driving system is used to obtain a response curve $F_{1}\left(V\right)$ and $F_{2}\left(V\right)$. The target gray-scale luminance is set to *R_0_*, and the permissible luminance oscillation range is Δ. Then, we can get Equation (2).

(2) $$F_{1}(V_{F})=R_{0}+\Delta$$

In Equation (2), the initial voltage $V_{F}$ can be obtained by testing EWDs. Then, $V_{F}$ is used to calculate $V_{E}$ by using $F_{3}\left(V\right)$. Hence, the stable driving voltage $V_{E}$ corresponding to *R_0_* can be obtained. Therefore, a gray-scale driving waveform can be formed by applying $V_{F}$, $V_{M}$, and $V_{E}$. The response time of the pixel can be accelerated by using the driving waveform, and it can reduce the luminance oscillation by using $V_{E}$ when *R_0_* is reached.

$F_{1}\left(V\right)$, $F_{2}\left(V\right)$ and $F_{3}\left(V\right)$ are the response curves of the oil in the pixel, and they all satisfy the Young–Lippmann principle [5]. When the maximum driving voltage value of the EWD is different, $F_{3}\left(V\right)$ is also changed. The hysteresis characteristic curve is simulated by the second fitting curve. Hence, Equation (3) is obtained.

(3) $$\{\begin{array}{c} F_{1}\left(V\right)=A_{1}V^{2}+B_{1}V+C_{1}\\ F_{2}\left(V\right)=A_{2}V^{2}+B_{2}V+C_{2}\\ F_{3}\left(V\right)=A_{3}V^{2}+B_{3}V+C_{3} \end{array}$$

According to Figure 4, Equation (4) can be obtained.

(4) $$\left\{ \begin{array}{l} F_{2}(V_{0})=F_{3}(V_{0})\\ F_{1}(V_{F})=F_{3}(V_{F}) \end{array}\right.$$

In Equation (3), $C_{3}$ is a constant, $F_{3}\left(V_{MAX}\right)$ and $F_{2}\left(V_{MAX}\right)$ are similar when $V_{F}=V_{MAX}$, and then, Equation (5) is obtained.

(5) $$C_{3}=C_{2}$$

The parameters of $F_{3}\left(V\right)$ can be obtained by using Equation (3)–(5), as shown in Equation (6).

(6) $$\left\{ \begin{array}{l} A_{3}=\frac{R_{MIN}V_{F}-R_{CUR}V_{0}}{(V_{0}-V_{F})V_{F}V_{0}}+\frac{C_{2}}{V_{F}V_{0}}\\ B_{3}=\frac{(R_{MIN}-C_{2})V_{F}^{2}+(C_{2}-R_{CUR})V_{0}^{2}}{V_{F}^{2}V_{0}-V_{F}V_{0}^{2}}\\ C_{3}=C_{2} \end{array}\right.$$

Then, the $F_{3}\left(V\right)$ is obtained, as shown in Equation (7).

(7) $$F_{3}(V)=\frac{R_{MIN}V_{F}-R_{CUR}V_{0}}{(V_{0}-V_{F})V_{F}V_{0}}+\frac{C_{2}}{V_{F}V_{0}}V^{2}+\frac{(R_{MIN}-C_{2})V_{F}^{2}+(C_{2}-R_{CUR})V_{0}^{2}}{V_{F}^{2}V_{0}-V_{F}V_{0}^{2}}V+C_{2}$$

The driving voltage at the target gray-scale *R_0_* is shown in Equation (8).

(8) $$V_{E}=\frac{-B_{3}\pm \sqrt{B_{3}^{2}-4A_{3}\left(C_{3}-R_{0}\right)}}{2A_{3}}$$

The main factors affecting the splitting of EWDs are the thickness of the oil center in the pixel and the shape of the driving waveform. The thickness of oil center in the pixel can be increased by adding an EPS to prevent oil from splitting, and the rising curve of the driving waveform can be output by the storage capacitance of the TFT, such as Equation (9).

(9) $$V\left(t\right)=U_{S}\left[1-e^{-\frac{t}{RC}}\right]$$

(10) $$\frac{dV}{dt}=\frac{U_{S}}{RC}e^{-\frac{t}{RC}}$$

*U_S_* represents VGH signal of the source IC, *R* represents the ITO wiring resistance, and *C* represents the storage capacitor. Equation (10) is the slope of the voltage curve, and the slope can be realized by using the storage capacitor *C* and the ITO wiring resistance *R* [16].

## 3. Driving Platform Based on TFT-EWD

### 3.1. Experimental Platform

**Figure 5:**
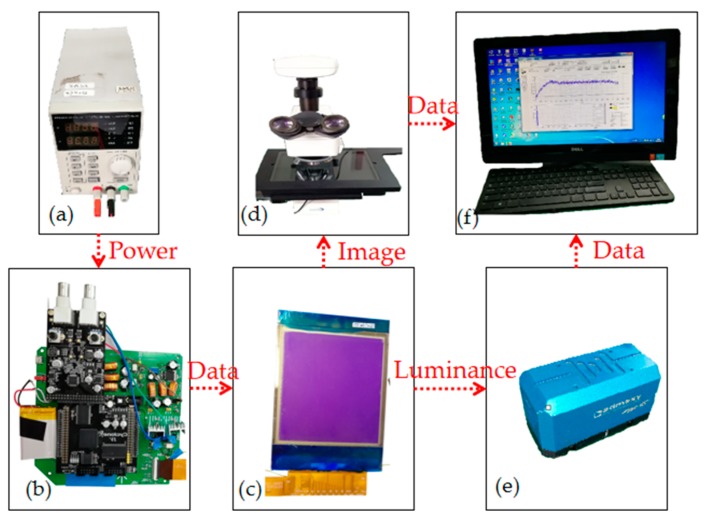
Experimental platform architecture.

In order to measure the display state of the EWD pixel in real time, a complete experimental platform is built, as shown in Figure 5. The main measuring device is an Admesy arg-45, which is a colorimeter developed by Admesy (Ittervoort, Netherlands). It has the characteristics of fast measuring speed and high measuring accuracy. In addition, there is a microscope (Shanghai CSOIF Co., Ltd, Shanghai, China) which can connect the computer to measure the state of the pixel graphically. Based on the TFT-EWD driving system, the output of amplitude–frequency mixed modulation driving waveform can be achieved. In Figure 5, (a) is the power adapter, (b) is the driving system based on TFT-EWD, (c) is an EWD panel, (d) is a microscope, (e) is the Admesy arg-45, and (f) is a computer. The light is emitted by the colorimeter with an angle of 45 degrees in the experimental platform. When the light is absorbed and reflected by the substrate in the pixel, the colorimeter can detect the intensity of the reflected light.

### 3.2. The Hardware Design of the Driving System

**Figure 6:**
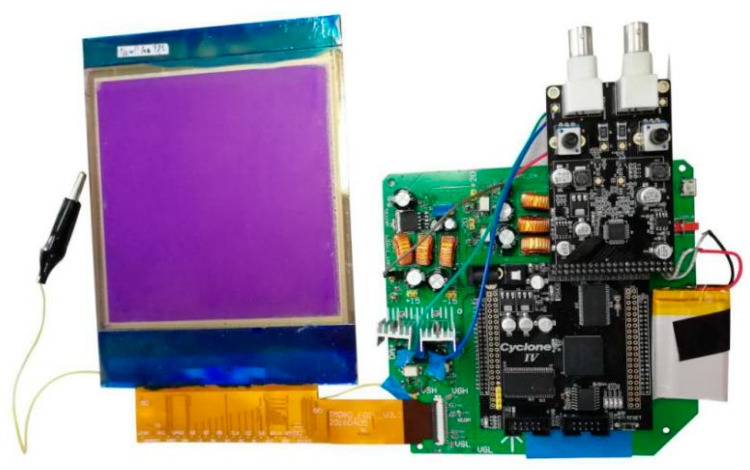
The driving system of the thin film transistor (TFT)-EWD.

In the design of the system platform, a high-speed parallel processing controller is used to implement the transmission of the display data and the adjustment of the driving voltage. In the hardware of the system, the function of timing controller is realized by a Field Programmable Gate Array (FPGA) chip, and the output control of driving waveform data can be completed. In addition, SOURCE and GATE chips are driven by the FPGA to control the gray-scale display of the TFT-EWD. A gray-scale conversion module is included in the FPGA, which converts the gray-scale data into the corresponding driving waveform. The system hardware also includes a high-speed DAC module which is also controlled by the FPGA. The Voltage Source High (VSH) and Voltage Source Low (VSL) signals of the SOURCE chip are controlled to realize voltage regulation by using the DAC module, and the purpose of adjusting driving voltage is then achieved. The physical diagram of the system is shown in Figure 6, and the parameter of the TFT-EWD is shown in Table 1.

**Figure 7:**
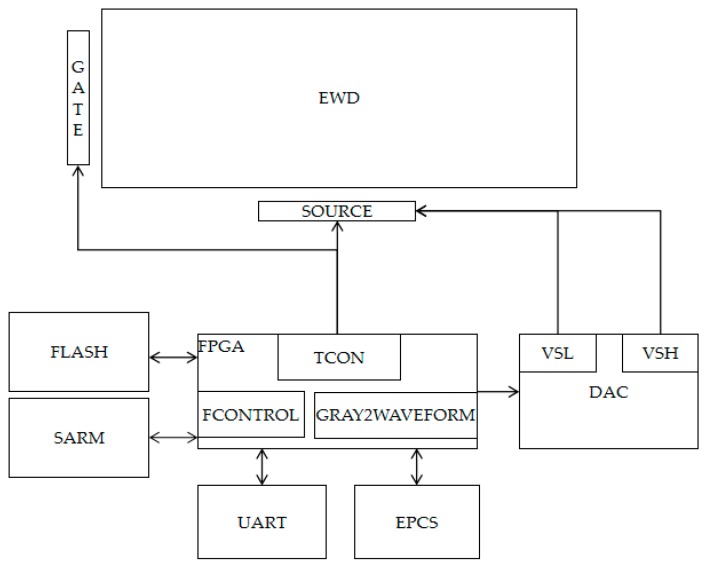
Driving system architecture of the TFT-EWD.

The fourth generation of Hurricane Series FPGA is used as the main control unit. Its internal logic clock can run in parallel on the basis of 200 MHz, and it has rich logic resources and Input- Output (IO) interfaces. The main processing principle of the system is that the gray-scale data, driving waveform, and configuration parameters which need to be displayed are transferred to a FLASH memory unit by an UART interface, and are saved. The driving waveform and configuration parameters of the FLASH memory unit are read when the FPGA is on power, and the program is initialized. Then, the gray-scale data in the FLASH is read by the FPGA, and the corresponding driving waveform data can be found by the program, and it can then be transferred to the Timing Controller (TCON) and the high-speed DAC module. The system architecture diagram is shown in Figure 7.

## 4. Driving Waveform Design of Amplitude–Frequency Mixed Modulation

**Figure 8:**
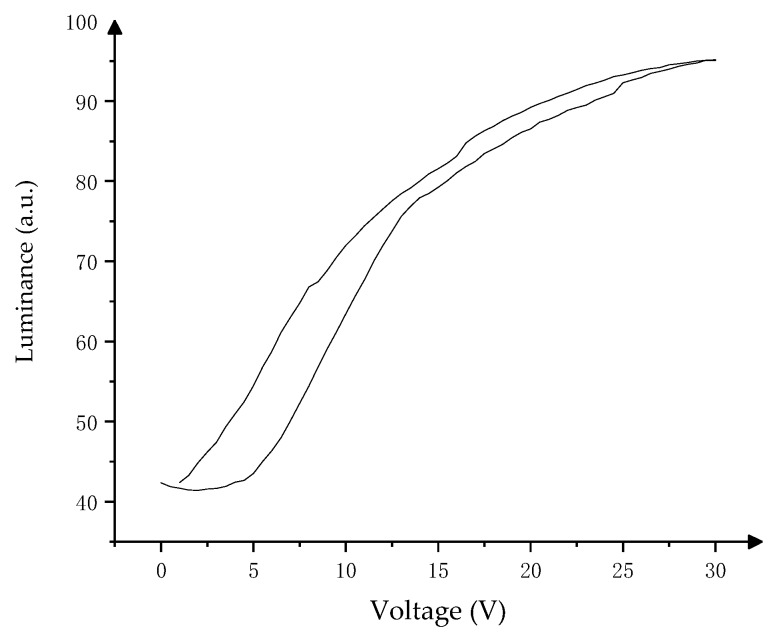
The hysteresis curve of an 8 inch TFT-EWD.

The VSL and VGL voltage of the source driver chip are adjusted by the high-speed DAC module to accurately control the frequency and amplitude in the driving waveform voltage. The EWD driving process is divided into the driving stage and the keeping stage. The driving stage is the driving waveform output stage, which is used to control the pixel luminance, and the keeping stage is used to control the oil distribution state to maintain a stable luminance. The driving stage in the driving waveform is mainly designed by amplitude–frequency mixed modulation principle. Driving data are obtained according to the hysteresis curve. The actual hysteresis curve is measured as shown in Figure 8. The constant driving voltage *V_E_* of the keeping phase is the last frame voltage in the driving stage.

For the 8 inch TFT-EWD in this paper, two curves of $F_{1}\left(V\right)$ and $F_{2}\left(V\right)$ in the hysteresis curve are fitted firstly, and the following results are then obtained by data fitting and error analysis, as shown in Equations (11) and (12), and the fitting similarity of functions is shown in Table 2.

(11) $$F_{1}\left(V\right)=-0.09831V^{2}+5.426V+18.67$$

(12) $$F_{2}\left(V\right)=-0.09831V^{2}+5.426V+18.67$$

**Figure 9:**
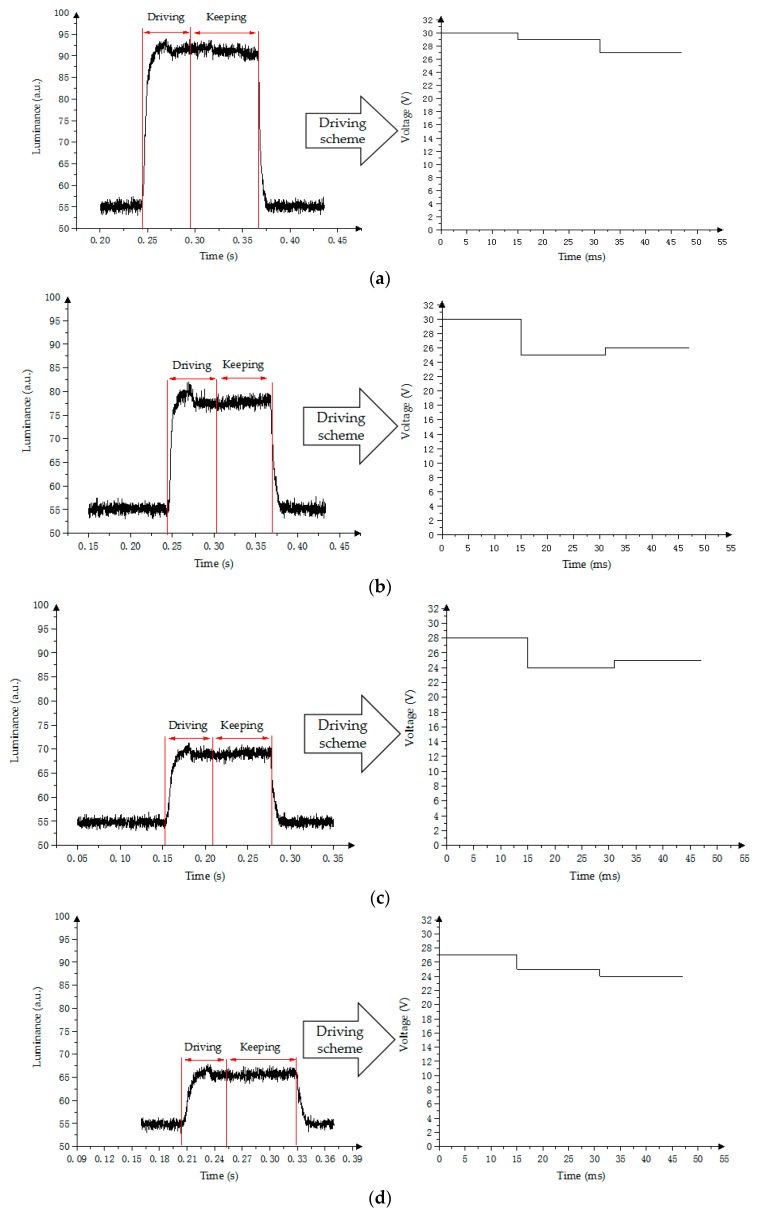
Amplitude–frequency mixed modulation waveform and its luminance response. (**a**) First gray scale; (**b**) Second gray scale; (**c**) Third gray scale; (**d**) Fourth gray scale.

In the testing process, the luminance of the target gray scale is set as *R_0_*, the permissible error range is set as Δ. The starting voltage is calculated according to the $F_{1}\left(V\right)$, $F_{3}\left(V\right)$ can be obtained using (4). The driving voltage *V_E_* corresponding to the curve can be obtained by *R_0_*. Therefore, the driving waveform of amplitude–frequency mixed modulation can be designed as follows: the driving voltage of the first frame is set as *V_F_*, the third frame is set as *V_E_*, and the luminance is stabilized at the target value *R_0_* by inserting adjusting voltage *V_M_* between the first frame and the third frame. The EWD is driven close to the expected luminance when the first frame is applied, as shown in Figure 9. The second frame is inserted to control the impact of the oil due to the voltage reduction [21]. The voltage which is applied in the third frame is used to adjust the luminance of the pixel for obtaining the target value.

In Figure 9, the maximum response time of the driving pixel to achieve stable gray scale by amplitude–frequency mixed modulation is 40 ms. A 4-level gray-scale display is used as an example, the luminance of the first gray scale is 95, that of the second gray scale is 83, that of the third gray scale is 72, and that of the fourth gray scale is 66.

## 5. Experimental Results and Discussion

### 5.1. Traditional PWM Scheme

In the traditional PWM scheme, one frame image is composed of multiple subframes, and the gray-scale control is carried out by adjusting the high and low voltage of each frame. Its theoretical essence is to drive the oil to achieve a specific distribution by applying the average effective voltage which is composed of multiple subframes. 

**Figure 10:**
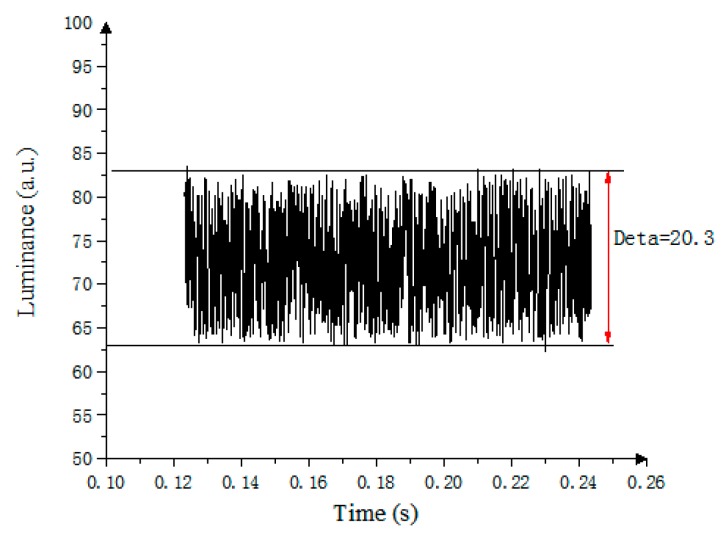
The oscillation characteristic of the oil in EWDs using PWM.

The lower the PWM frequency is, the stronger the oil oscillation is. As shown in Figure 10, the maximum oscillation amplitude of the fourth gray scale is Δ = 20.3. In the gray-scale display, in order to make the gray scale show a linear distribution for a better visual display effect, gamma correction technology was used to adjust the gray scale at all levels in the LCD to meet the linear distribution. However, in the PWM scheme, the gamma correction cannot be adjusted appropriately, which is one of the defects of the PWM scheme in EWD driver [15,17]. In addition, the PWM cannot accurately display multilevel gray scale. At present, TFT-EWD systems which are driven by the PWM are realized by using industrialized electrophoretic paper driver chips, but the refresh speed of the driver chips is less than 200 KHz. The relationship between the minimum screen refresh time *t* and the number of subframes *N* is shown in Equation (13):

(13) $$t\times N\leq \frac{1}{freq_{e}}$$

In Equation (11), $f_{req_{e}}$ is the flickering frequency which can be recognized by the human eye. Currently, the standard refresh frequency of LCD screen is 30 Hz, which is $f_{req_{e}}\leq 30$, and we can get Equation (14).

(14) t×N≤33.3

According to the parameters of industrialized electrophoretic paper driver chips and the EWD resolution, the minimum time *t* (t = 4.6ms, TFT-EWD is 8 inch) can be calculated, so the maximum number of subframes is 33.3/*t* (N = 7, TFT-EFD is 8 inch). Therefore, the PWM scheme meets the bottleneck when the multilevel gray scale is displayed in EWDs.

**Figure 11:**
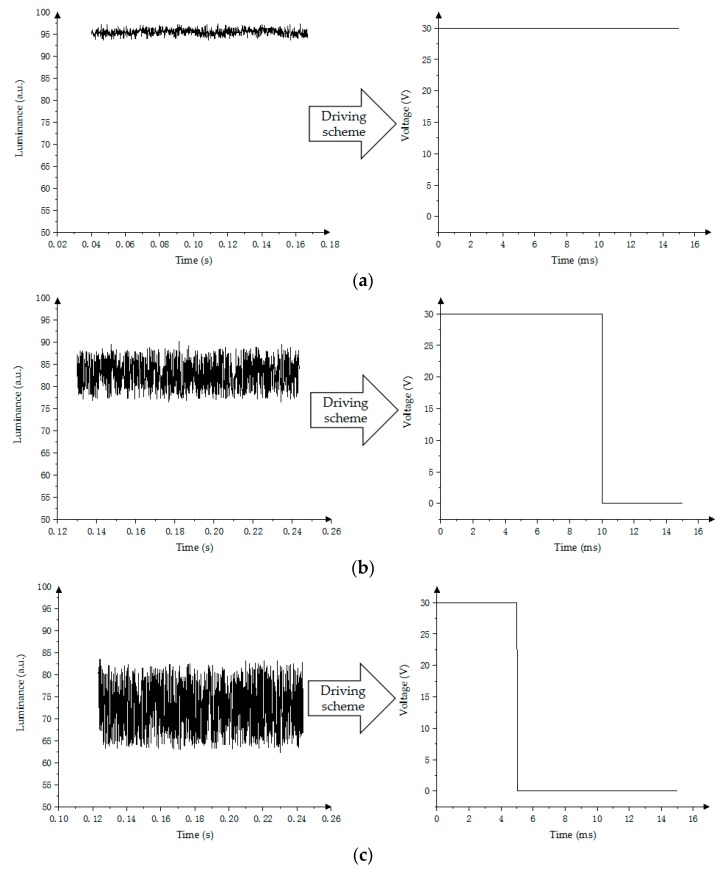

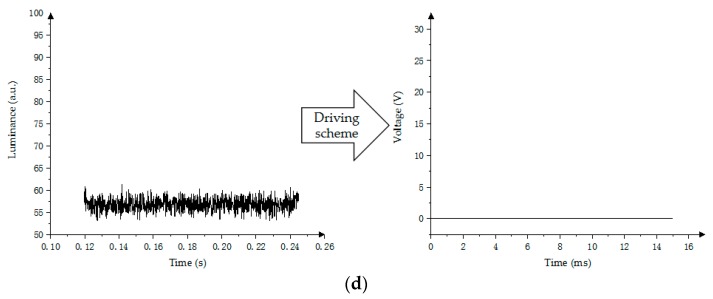
Four-stage driving waveform and its response curve by using PWM scheme. (**a**) First gray scale; (**b**) Second gray scale; (**c**) Third gray scale; (**d**) Fourth gray scale.

The PWM has been used to drive the EWD with four gray levels [15,17]. Real-time luminance data are measured by the Admesy device. As shown in Figure 11, the luminance of the first gray scale is 95, the second gray scale is 83, the third gray scale is 72, and the fourth gray scale is 57.

### 5.2. Gray-Scale Stability of the Amplitude–Frequency Mixed Modulation 

In order to verify the performance of the mixed modulation method, the gray-scale stabilization phases of the traditional PWM scheme and the amplitude–frequency mixed modulation are measured by the timing function of the Admesy colorimeter, and a test example of the fourth-stage gray scale is used in the experiment. The system platform includes an optical measuring instrument, an Admesy colorimeter. In the Admesy colorimeter, there is a standard light source for measuring the luminance of EWDs. The data measured by the Admesy colorimeter are displayed in real time by computer software.

**Figure 12:**
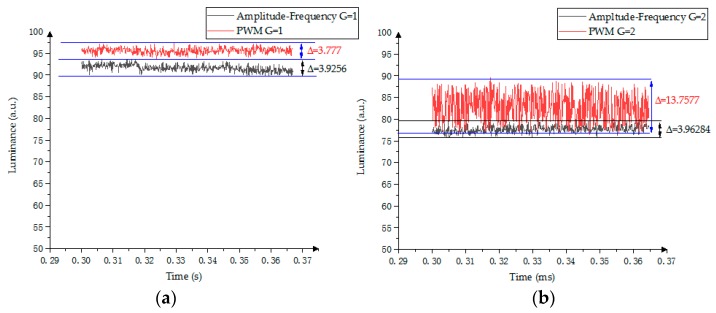

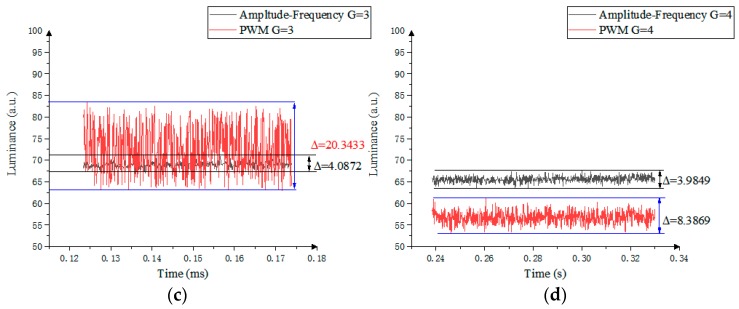
Gray-scale stability comparison between PWM modulation and amplitude–frequency mixed modulation. (**a**) First gray scale; (**b**) Second gray scale; (**c**) Third gray scale; (**d**) Fourth gray scale.

In the amplitude–frequency mixed modulation, the stable phase (keeping phase) is driven by DC voltage, and the oil oscillation is within 3.9256 < Δ < 4.0872, as shown in Figure 12. In the PWM scheme, the range of oil oscillation measured during the stabilization stage is within 3.777 < Δ < 20.3433. In the gray scale of G = 2 and G = 3, the longer the low voltage time is, the greater the oil oscillation when the driving frequencies are the same as each other.

**Figure 13:**
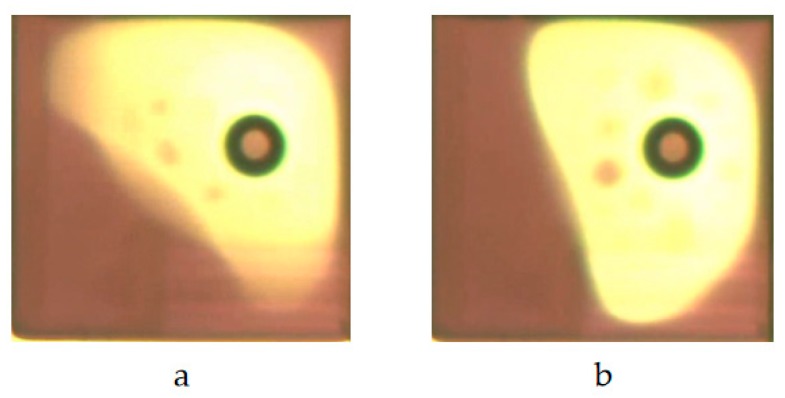
Stable image of pixel when the gray scale is 3. (**a**) PWM scheme; (**b**) Amplitude–frequency mixed modulation.

A computer is connected to a microscope records and saves the oil motion status parameters in a single pixel in real time. In Figure 13a, the stable state parameters of G = 3 (third gray scale) are obtained when the oil is driven by the PWM scheme, and the stable state parameters of G = 2 (second gray scale) is shown in Figure 13b when the oil is driven by the amplitude–frequency mixed modulation. It is found that the PWM driver produces serious oscillation on the edge of the oil, which leads to blurring at the edge of the oil.

**Figure 14:**
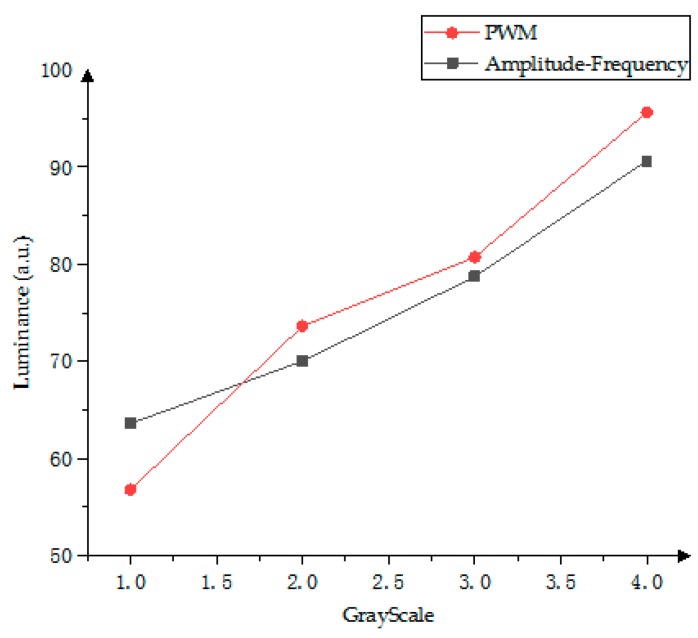
Luminance linear comparison of gray scale between the PWM scheme and the amplitude–frequency mixed modulation.

In Figure 14, black line is the gray-scale luminance distribution of the amplitude–frequency modulation and red line is the gray-scale distribution of the PWM modulation. The gray-scale luminance interval driven by the amplitude–frequency modulation can be more uniform, which can enhance the visual perception of the human eye.

### 5.3. Response Time of the Amplitude–Frequency Mixed Modulation

**Figure 15:**
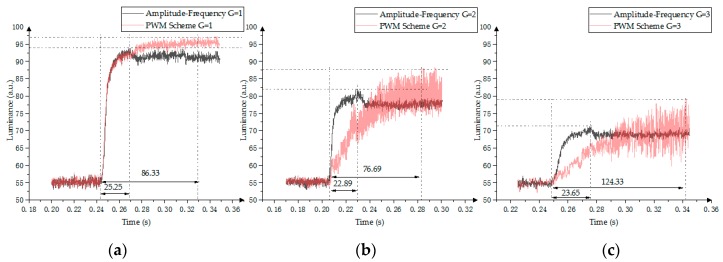
Response time comparison between the PWM scheme and the amplitude–frequency mixed modulation. (**a**) First gray scale; (**b**) Second gray scale; (**c**) Third gray scale.

The response time of the amplitude–frequency mixed modulation is shown in Figure 15. The red line represents the PWM scheme, and the black line represents the amplitude–frequency mixed modulation driver. The driver voltage is 0 V in the PWM scheme when G = 4 (fourth gray scale), so there is no response time comparison at this time. The amplitude–frequency mixed modulation response time range is 22.89 ms < *t* < 25.25 ms, and the PWM response time range is 86.33 ms < *t* < 124.33 ms. Hence, compared with the PWM scheme, the amplitude–frequency mixed modulation can shorten the response time by 70%.

## 6. Conclusions 

The gray-scale display and response time of traditional PWM scheme for driving EWDs are analyzed in this paper. The PWM scheme has theoretical limitations and cannot display more effective gray scales than existing studies. Therefore, we proposed an EWD device driver system platform with a DAC for regulating source voltage values. The precise gray-scale controller of the EWD is realized by using the amplitude–frequency modulation method. Therefore, the response time of the EWD is improved and dynamic images with rich gray-scale information can be displayed, which can improve the visual effect of EWDs.

## Figures and Tables

**Table 1:** Parameters of the TFT-EWD.

Panel Size	Resolution	Pixel Size	Pixel Wall Height	Oil Color	Driving Voltage
8 inch	720 × 720	180 μm × 180 μm	12 μm	Cyan	0–30 V

**Table 2:** The fitting similarity of hysteresis curves.

Fitting Similarity of the Function	Variance SSE (Sum of Squares for Error)	Coefficient of Determination R-square	Adjusted R-square	Standard Deviation RMSE (Root Mean Squared Error)
$F_{1}\left(V\right)$	104.2	0.9904	0.99	1.489
$F_{2}\left(V\right)$	34.47	0.9978	0.9977	0.7845
